# The role of the big geographic sort in online news circulation among U.S. Reddit users

**DOI:** 10.1038/s41598-023-33247-3

**Published:** 2023-04-25

**Authors:** Lia Bozarth, Daniele Quercia, Licia Capra, Sanja Šćepanović

**Affiliations:** 1 University of Michigan, Ann Arbor, USA; 2 Bell Labs, Cambridge, UK; 3 CUSP, Kings College London, London, UK; 4 University College London, London, UK

**Keywords:** Complex networks, Computer science

## Abstract

Past research has attributed the circulation of online news to two main factors—individual characteristics (e.g., a person’s information literacy) and social media effects (e.g., algorithm-mediated information diffusion)—and has overlooked a third one: the critical mass created by the offline self-segregation of Americans into like-minded geographical regions such as states (a phenomenon called ‘The Big Sort’). We hypothesized that this latter factor matters for the online spreading of news not least because online interactions, despite having the potential of being global, end up being localized: interaction probability is known to rapidly decay with distance. Upon analysis of more than 8M Reddit comments containing news links spanning four years, from January 2016 to December 2019, we found that Reddit did not work as an ‘hype machine’ for news (as opposed to what previous work reported for other platforms, circulation was not mainly caused by platform-facilitated network effects). Rather, news circulation in Reddit worked as a supply-and-demand system: news items scaled linearly with the number of users in each state (with a scaling exponent $\beta$  $\approx 1$, and a goodness of fit $R^2\approx 0.95$). Furthermore, deviations from such a universal pattern were best explained by state-level personality and cultural factors ($R^2\approx \{0.12, 0.39\}$), rather than socioeconomic conditions ($R^2\approx \{0.15, 0.29\}$) or political characteristics ($R^2\approx \{0.06, 0.21\}$). Higher-than-expected circulation of any type of news was found in states characterised by residents who tend to be less diligent in terms of their personality (low in conscientiousness) and by loose cultures understating the importance of adherence to norms (low in cultural tightness). Interestingly, the combination of those factors with low levels of education was then associated with the circulation of a particular type of news, that is, misinformation. These results suggest that online interactions are geographically bounded and, as such, news circulation cannot be studied purely as an Internet phenomenon but should be grounded into a user’s offline cultural environment, which has become increasingly segregated over the decades, and is admittedly hard to change.

## Introduction

Past research has attributed the circulation of online news to two main classes of factors. The first class includes individual characteristics such as a person’s personality and culture, education attainment, and political-leaning^1–9^, often reinforced by confirmation bias^10,11^. For example, users highly driven by self-presentation (personality) share more news^12,13^, and political leaning affects the type of political news users share^14^. Further, those with lower information literacy were observed to be more likely to spread misinformation^15^.

The second class of factors has to do with the ways social media are engineered to work as a “Hype Machine”^16^. For instance, existing social media platforms’ “friends suggestion algorithms”—which tend to disproportionately recommend friends of friends who likely share similar behaviors and beliefs—have amplified the online clustering of individuals into homophilous communities. Users were also observed to be more likely to team up with like-minded others, which is commonly known as the echo chamber or filter bubble effect^17,18^. Another platform-amplified feature is affect. Platform algorithms were observed to preferentially recommend emotionally salient and polarizing content to boost user engagement and content sharing^19,20^. Prior studies demonstrated that these small and densely connected online communities had significantly increased the size, depth, and speed of online spreading^21^. Indeed, online news circulation follows news cycles^22^, influences social media users^23^ who, in turn, influence each other^24,25^, even beyond informational purposes^13^, creating a news distribution system that goes beyond a simple supply-and-demand system^26^.

There is, however, a third overlooked factor: the offline self-segregation of Americans into like-minded communities such as geographic states, a phenomenon which Bill Bishop dubbed as “The Big Sort”^27^. Work by Bishop and others has illustrated that people in the U.S. have been increasingly choosing to live in neighborhoods populated with others who are just like themselves in values and beliefs. Furthermore, this sorting has resulted in geographical regions (e.g., states) with distinct lifestyle and culture^28–30^, political ideology^31^, and even personality^32–34^. As an example, work by Rentfrow et al.^33^ showed that the states of Utah and New York are the most and least agreeable among all the states, respectively. South Carolina is the most conscientious, and Maine the least. Similarly, Mississippi has the most restrictive cultural and social norms, whereas California has the most loose^33^. Furthermore, states’ personality and culture are indicative of their voting patterns^32^. Previous research found that the circulation of physical newspapers follows readership interests^35^. Moreover, each newspaper matches its political slant to its readers’ slant^36^. The process of Americans geographically sorting themselves over the past four decades into homogeneous communities still continues. Thus far, it is unclear whether it has had any impact on *online* news circulation.

To ascertain that, we examined the geographical circulation of news on Reddit, a popular online content aggregation and discussion website. We chose Reddit for our analysis given that it has one of the most comprehensive publicly available archived datasets (available under pushshift.io). Reddit consists of many communities (or areas of interest) called subreddits that function akin to online forums. Users can make public posts on these subreddits and others can then comment on the original posts. For instance, a user can post a news article about Covid-19 on the subreddit r/news, and others can then discuss the article with each other. Unlike social media platforms such as Twitter and Facebook, Reddit is an anonymous platform without the concept of ‘friends’. This anonymity in Reddit might have the advantage of removing the typical social pressure mechanism of circle-of-friend platforms like Facebook or Twitter. Therefore, Reddit is the ideal platform to single out and study geographic factors and their influence in news circulation.

## Data

### Reddit data

**Figure 1:**
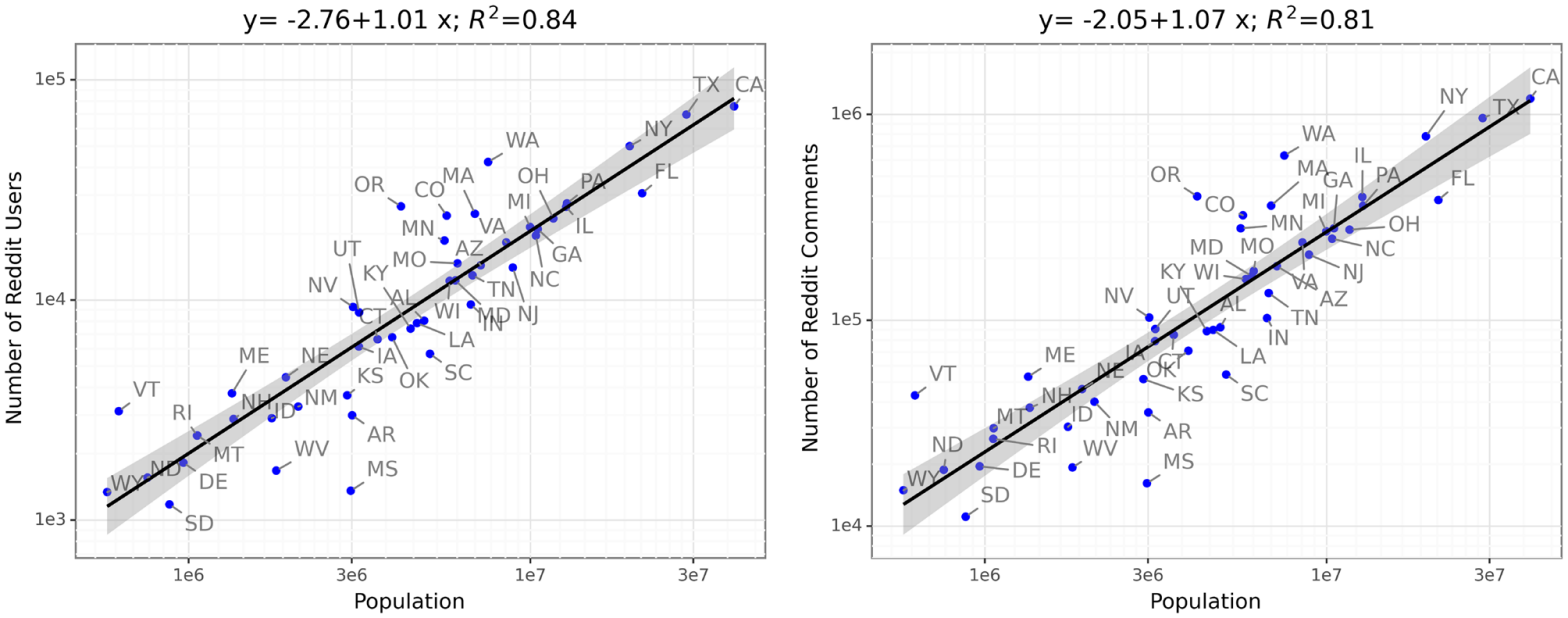
Reddit users and comments per state: The *x*-axis denotes each state’s population (logged) and the *y*-axis is the number of Reddit users/comments from each state. We see that the number of Reddit users/comments scaled linearly with the population ($\beta =1.01/1.07$), with an $R^2=0.84/0.81$.

We used Pushshift’s^37^ publicly available comments dataset from January 2016 to December 2019. This dataset contained all comments from all public and quarantined subreddits. We then used the method from Balsamo et al.^38^ to assign users to their geographical location. Specifically, we first identified a list of 2.87K subreddits that can be matched to one of the U.S. states (e.g., r/seattle, r/california). Then, for each user who had posted at least once in these subreddits, we assigned the user to the corresponding U.S. state. Note that if a user had posted in multiple states, we assigned the user the state with the majority of posts. As a result, 82.4% of users had only posted in a single state, and 95.2% of users had posted in at most 2 states. Finally, only 3.8% of users were not assigned a state due to not having a majority state. We identified approximately 3M users who were located in one of the 50 U.S. states. The correlation between a state’s population and its number of Reddit users is shown in Fig. 1. We saw that the number of Reddit users per state scaled linearly with the state’s population ($\beta =0.99$). Additionally, approximately 1.4 billion (or 35%) comments on Reddit can be mapped to a user in one of the 50 U.S. states. From these 1.4B comments, we identified a total of 8.23M (0.6%) comments containing news links (as URLs). We then classified a Reddit comment as either *reputable*, *fake*, or *low credibility* based on the *domain* that the news URL pointed to, using the groundtruth labeling procedure described next.

**Table 1:** Summary statistics for news comments.

News_type	Unique_comments	Unique_user	Unique_news_site	Unique_urls	Top_news_sites
Fake	116212	45485	933	60754	breitbart.com, dailywire.com, thegatewaypundit.com
Lowcred	536701	160146	1801	264010	dailymail.co.uk, washingtonexaminer.com, dailycaller.com
Reputable	7645044	717198	5221	3319213	nytimes.com, washingtonpost.com, wsj.com

These comments are Reddit posts that contain links to news articles of three types.

### Website groundtruth labels

We compiled a list of news websites (or domains) from various sources widely used in researching online news circulation^39^. Each news site was then labelled as one of three types—*fake, lowcred*, or *reputable*—as follows.

#### Reputable

We used three sources to compile a list of reputable news sites: Vargo et al.^40^, Alexa (alexa.com), and Media Bias/Fact Check (mediabiasfactcheck.com). This resulted in 8.9k total reputable news sites.

#### Fake

Based on a detailed meta-review in related work^39^, we compiled a list of questionable news sites from 5 existing sources: Zimdars list^41^, Media Bias/Fact Check, PolitiFact^42^, the Daily Dot^43^, and Allcott et al.^44^. By using the descriptions and granular labels of each of the five sources, we categorized a domain as *fake* if it had routinely published completely fabricated news articles. There were a total of 933 unique fake news sites across all five sources.

#### Lowcred

Unlike *fake* news sites, low-credibility news sites publish articles with mixed factualness rather than completely fabricated content. We included domains that were described by the previous 5 sources as unreliable, hyperpartisan, clickbait, rumor, pseudoscience, and conspiracy sites, ending up with a total of 1801 low-credibility news domains.

**Table 2:** List of state-level attributes.

Category	Variable name	Description
Personality and culture	Openness	Imaginative, spontaneous
	Conscientiousness	Disciplined and careful
	Extraversion	Social and fun-loving
	Agreeableness	Trusting and helpful
	Neuroticism	Anxious, pessimistic
	Cultural_tightness	Restrictive social norms and punishments for deviance
Socio-economic	density	Population density (proxy for urbanization) 2019
	Gdp	State’s gdp per capita 2019
	Minority	Percentage of person of color 2019
	No_highschool	Percentage of population without a high school diploma 2019
	Population	State population on 2019
Political	Political	Political engagement score
	Republican	Percentage prefer republican subtract percentage prefer democrat
	Swing_state	Binary score of 0 (not swing state) or 1 (swing state)
Platform	Adoption	Adoption rate of reddit

Using the compiled domain credibility lists, we labelled individual news articles with corresponding domain labels. Hence, we attributed misinformation at the level of the publisher (i.e., domain) and not at the level of the individual news article, which would be more precise. Nevertheless, the approach we took is widely used in misinformation studies^39^. Additionally, while our lists of news sites are widely popular in researching misinformation, prior work had highlighted that the different lists had been created using varying labeling procedures^39^. As such, we included additional steps detailed in Supplementary Material to validate our news site classification approach. Briefly, we compared our labels (*fake, lowcred*, and *reputable*) to trustworthiness scores of news sites provided by professional fact-checkers^45^, and observed that reputable news sites had the highest average trustworthiness score (0.66), followed by low-credibility news sites (0.10), and finally fake news sites (0.02), suggesting that our labels were well aligned with the ratings of professional fact-checkers.

### Classification of news comments

The circulation of news on Reddit (Table 1) amounts mainly to reputable content: 7.6M (93%) comments contained reputable news articles, while only 116.2K contained fake news articles. We also observed that reputable news sites attracted, on average, only 36 Reddit comments, low-credibility 26, and fake 8. Those low average values are due to the frequency distribution of the number of comments per news site being skewed: most news sites attract a few comments only, while a few attract most comments (e.g., approximately one-fifth of all fake news comments contained URLs from breitbart.com). To then ascertain that our localization procedure did not select a specific type of user but selected a set representative of the general user population, we compared the 3M users with assigned locations to another 3M users without locations. We observed that the average numbers of comments posted by users of the two groups were comparable, with just a small difference: 1.7% of all geotagged users had posted at least 1 comment containing fake news URLs, whereas only 0.6% of non-geotagged users did. This difference can be explained by non-geotagged users being less invested in U.S. news as, on average, they are less likely to all be from the U.S.

**Figure 2:**
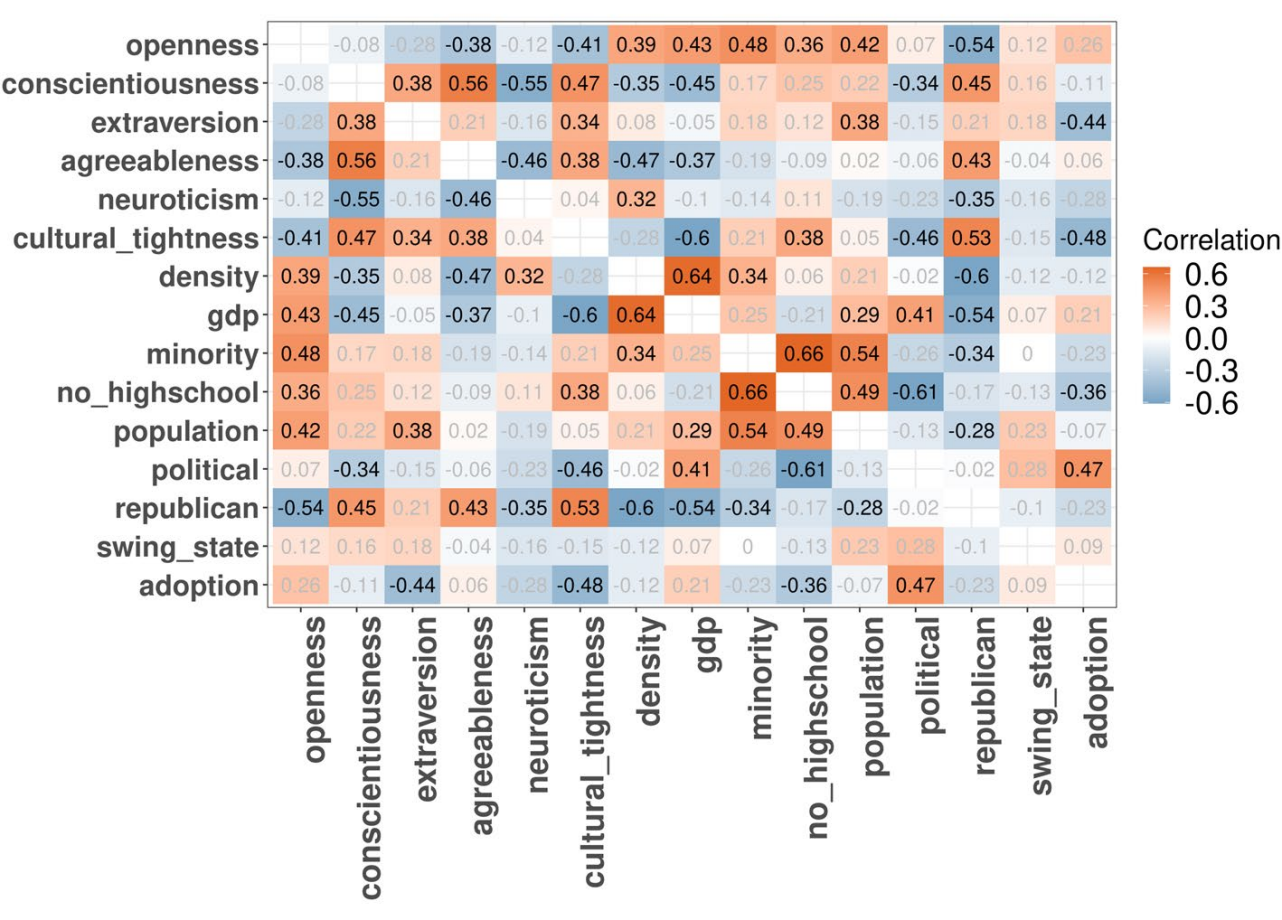
Cross-correlation between state-level factors. Statistically insignificant correlations (*p*-value$\ge 0.05$) are grayed out. The matrix was created using version 0.92 of the following R package https://cran.r-project.org/web/packages/corrplot.

### State-level attributes

We included the following state-level attributes that were shown by prior studies to be indicative of individual and community’s tendency to share misinformation^2,5,46^. These attributes were categorized into personality and cultural factors, socio-economic conditions, and political attributes (Table 2).

#### Personality and culture

Prior work had observed significant individual-level associations between personality/culture and circulation of misinformation^2,6,47,48^. For instance, individuals scoring high in conscientiousness are significantly less likely to spread false content^2^. Similarly, a lower level of extraversion is associated with a higher discernment of misinformation^49^. One of the most commonly used *personality* tests is the Big Five test, which measures five main traits (abbreviated as OCEAN) ^50,51^: Openness (creative and open-minded), Conscientiousness (organised and responsible), Extraversion (sociable and energetic), Agreeableness (compassionate and compliant), and Neuroticism (anxious and emotionally unstable). We used the test results of 1.69M respondents in the U.S.^33^. Analyses of these results found the traits to differ across states^34,52^, and to influence a variety of aspects, including information and knowledge sharing preferences^53–55^. Another trait related to the task at hand (circulation of information) is *cultural tightness*. This measures the propensity of a society to conformity^56^, and has been associated with a variety of aspects concerning information sharing practices, such as digital engagement, knowledge sharing, and acceptance of diverse opinions^57–61^. This latter variable reflects also the propensity of holding adherence to norms in high regard^59^, and might well be hindering the spreading of misinformation.

#### Socio-economic

Some socioeconomic factors are indicative of an individual’s political knowledge, information literacy, and tendency to consume and diffuse news or misinformation^5,44,46^. As an example, individuals who are socio-economically well-off tend to have more political knowledge^62^, which is associated with having a better ability in telling apart factual news from misinformation^46^. Overall, in terms of socio-economic indicators, we included five variables available from the 2019 American Community Survey: population (*population*); population density as a proxy for urbanization (*density*); percentage of population over 25 years old without high school diploma (*no_highschool*); percentage of person of color (*minority*); and gdp per capita (*gdp*).

#### Political

The extensive literature review^1^, found that news sharing is ‘a specific kind of participatory behavior that is dependent on people’s [...] political interests’ and that content featuring politics, government, or economics is increasingly spread during the heightened political activity^63^. As such, it is valuable to consider environmental influences, such as political participation and leaning on general news sharing^1,63^. Specifically for fake news, it is repeatedly found to be politically driven and is more likely to be consumed and shared by conservative-leaning individuals and online communities^5,44,46,64–66^. Therefore, we postulated that states’ political attributes would be among the most indicative of the states’ tendency to circulate particular news and, especially, misinformation, and consequently included three political attributes: percentage gap between the population leaning towards the Republican party and that leaning towards the Democratic party (*republican*) provided by the 2016 Gallup Poll; whether a state was a battleground state during the 2016 presidential election or not (*swing_state*) provided by the Center for Politics; and the political engagement score (*political*) from^67^, which was calculated using the weighted sum of multiple metrics (i.e., percentage of registered voters, total political contribution, and percentage of residents who participated in local political) provided between 2016 and 2019 by the American Community Survey, the U.S. Census Bureau, the Center for Responsive Politics, and Ballotpedia.

To those socio-economic attributes, we added a state’s Reddit adoption rate as a control varaible. That is because online news circulation might well be explained by online adoption rates, which, in turn, happened to be correlated with some of the socio-economic attributes in our case (Figure 2): negatively with *extraversion, cultural_tightness*, and *no_highschool*, and positively with *political*. In other words, states that are social, culturally restrictive, and have low education attainment have fewer-than-expected users on Reddit.

## Methods

### Scaling laws of news circulation

To study circulation within states, we resorted to urban science research in the area of complex systems^68,69^. Such work has shown that a variety of urban measures such as number of patents and income are power-law functions of population size^69,70^. Yet, we do not know whether that is the case for news circulation online: critics might rightly say that the process of online circulation may have little to do with a user’s offline conditions or may be just “too complex” to be subject to laws.

To investigate the relationship between news circulation and population size, we used a methodology that was put forth by Bettencourt et al.^69^. Say that *Y* denotes circulation within a state, then this power-law dependency translates into saying that $Y = constant \cdot N^\beta$. By then taking the log of both sides, we obtain: $\log (Y) = \beta \cdot \log (N) + constant$, where *N* is the population size, *constant* is a normalization constant, and $\beta$ is the so-called *scaling exponent*. Typically, the values of this scaling exponent are grouped in three ranges:

- $0.8 > \beta$ (*sublinear*) is found for material quantities displaying *economies of scale* (e.g., infrastructure);
- $0.8\le \beta < 1.1$ (*linear*) is found for individual human needs (e.g., jobs, houses);
- $1.1 \le \beta < 1.3$ (*superlinear*) is found for measures reflecting wealth creation and innovation with *increasing returns*, which are typically associated with the intrinsically social nature of large cities (e.g., number of patents, number of successful startups).

### Three types of news

Since the number of Reddit users alone could explain a great portion of the variance in the online circulation of the three types of news, we used the following approach to separate the impact of platform adoption and the characteristics of a state. Given a news type $s \in \{lowcred, fake, reputable\}$ and state *i*, let $\beta ^s$ be the scaling exponent for news type *s*, and $\beta _0^s$ the corresponding intercept term, $f_{s,i}$ denote the total number of news items of type *s* posted by users from *i* (in log value), and $N_i$ be the number of users in state *i* (in log value). We then run the simple regression $f_{s, i}= {\beta _0^s} + \beta ^s N_{i} + \varepsilon _{s,i}$ to determine the residual $\varepsilon _{s, i}$, which we call the *Residual Circulation(s,i)* score of state *i* for the news type *s*. This is the portion of the circulation of news of type *s* in a state *i* that is not explained by the number of users in *i*. Next, we took that residual and run the following model:

1 $$\begin{aligned} {Residual}\ Circulation({s,i}) = \beta _0^\prime + \beta _1^\prime * v_{1} + \beta _{2}^\prime * v_{2} \cdots + \beta _{n}^\prime * v_{n} + \varepsilon ^{\prime }, \end{aligned}$$

where $v_1$, $v_2$, and $v_n$ are the predictors listed in Table 2. Note that all variables were standardized with *z*-scores to make regression coefficients easier to interpret. For comparability’s sake, in addition to this circulation metric based on the residual, we also used the average number of news comments as as an alternative metric (i.e., *Circulation*(*s*, *i*) was calculated as the average number of comments containing URLs to news type *s* posted by Reddit users from state *i*), and reported the results in Supplementary Material; both metrics showed comparable results.

## Results

**Figure 3:**
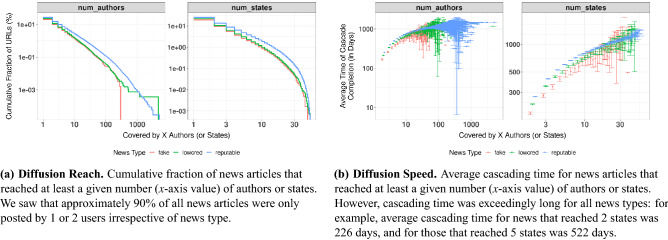
Diffusion of the three types of news (news type classification is based on domains) in Reddit.

### The role of platform-facilitated news diffusion

For each type of news (i.e., reputable, low-credibility and fake), we computed the cumulative fraction of articles that reached at least a given number of authors or states (Fig. 3a). We observed that geographical diffusion is rare on Reddit. More specifically, 74.8% of all reputable news articles were only posted by a single user who was located in the U.S., and 86.7% by at most 2 users. The values were comparable for fake and low-credibility news. Additionally, the number of news URLs that were posted in 5 or more states was only 209.7K for (6.3% of) reputable news comments, 11.0 K for (4.8% of) low-credibility ones, and 2.23K for (4.2% of) fake ones. Furthermore, we also observed that the time gaps between the comments were lengthy (Fig. 3b). For example, for all news URLs that reached exactly 5 states (only 6% of news had reached 5 or more states), the average cascading time was over a year. We also ran analysis using the median cascading time, and results were similar. In sum, our results demonstrate that circulation of news on Reddit is unlikely to be a function of diffusion, and there are several likely explanations for it. First, to reduce content duplication, Reddit moderators typically discourage users from reposting the same content on the same subreddit or even on different subreddits^71^. Another explanation could be geographical segregation. As the literature showed for platforms other than Reddit^72,73^, online users who live far away could be less likely to interact with each other, thus reducing out-of-state news circulation in the case of Reddit. Our data allowed us to test this latter explanation, and we did so next.

#### The role of geographical proximity

**Figure 4:**
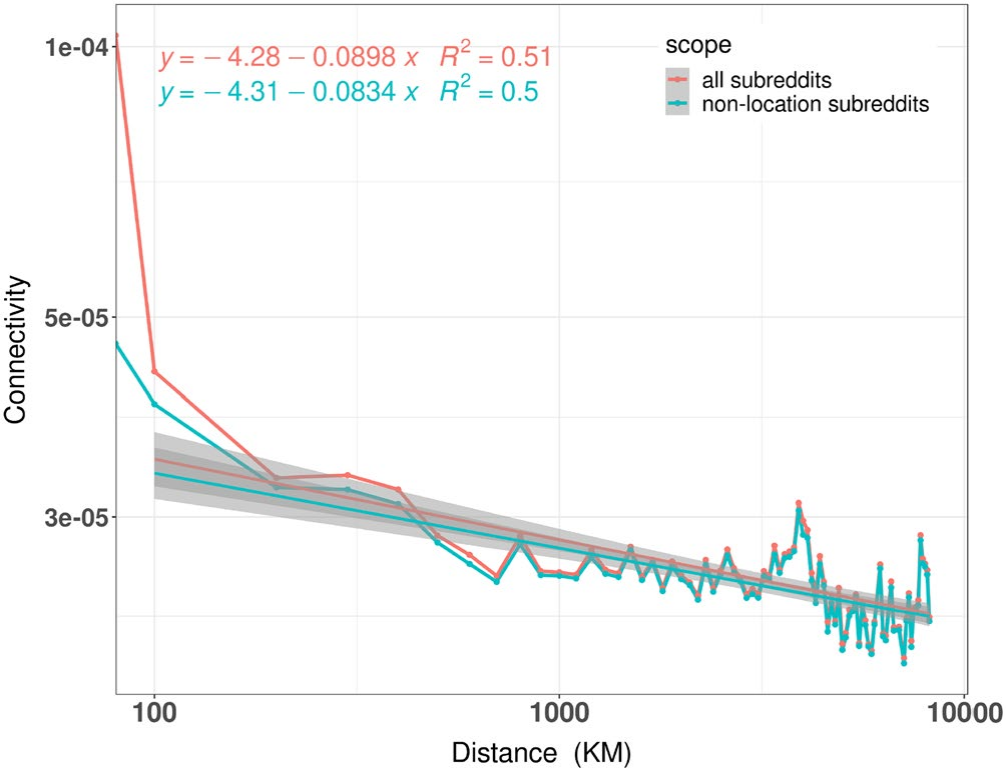
Geographic distance *vs.* *Connectivity*. The *x*-axis denotes the geographical distance between states’ centers and the *y*-axis is the probability that a pair of users with *x* distance apart offline had interacted with each other on Reddit. Finally, the color denotes the scope of interaction. We surprisingly saw that even for subreddits without an inherent geographical affiliation, users still preferred to interact with others of closer geographical proximity.

To test the extent to which online interactions are impacted by geographical distance, we adopted a metric from related work^72^. More specifically, we first generated a user-to-user comment network in which an edge exists between a pair of users, if one user had commented on the other’s comment/post^74^. The resulting network was unidirectional and weighted. We then computed the probability of having had an interaction, denoted as $Connectivity_d$, between a pair of users who are at *d* physical distance apart (measured in km). The distance *d* between a pair of users was calculated as the distance between the geographical centers of the states that the pair resided in (users from the same state have $d=0$). Mathematically, for a fixed distance *d* where $d=\{0 km, 100 km, 200 km, 300 km...\}$, we calculated $Connectivity_d$ as:

2 $$\begin{aligned} Connectivity_{d} = \frac{|comments_{{i,j}}|_d}{\frac{1}{2}*N_d*(N_d-1)}, \end{aligned}$$

where $N_d$ is the total number of users that were approximately *d* distance apart offline, and $|comments_{{i,j}}|_d$ is the total number of unique pairs of users who lived *d* distance apart and who interacted on Reddit (this number is the corresponding weight on the user-to-user comment network). The denominator $\frac{1}{2}*N_d*(N_d-1)$ is the total number of possible user pairs at distance *d*. In other words, given *d*, $Connectivity_d$ is the number of user pairs that interacted with each other normalized by the total number of possible user pairs. We then plotted the logged $Connectivity_d$ in relation to the logged physical distance *d* in Fig. 4(red line). Consistent with prior work^72^, we found that $Connectivity_d$ rapidly decreases with *d*. For instance, users located approximately 100km apart had $4.35\textrm{e}{-5}$ probability of interacting with each other via comments. Whereas, the probability decreased to $2.6\textrm{e}{-5}$ for users located 1000km apart. In other words, geographic proximity increases the probability of interacting (i.e., users located closer in physical distance are more likely to interact with each other): indeed, the probability of interacting is highest for users of the same state ($1.02\textrm{e}{-4}$) as it is one order of magnitude higher than the out-of-state’s probability ($\ge 2.6\textrm{e}{-5}$). Next, to ensure that our observation was not primarily driven by interactions on location-specific subreddits (e.g., r/seattle, r/california), we also limited the *scope* of interaction to non-location subreddits. To that end, we updated the definition of $|comments_{{i,j}}|_d$ to be the number of unique pairs of users who lived *d* distance apart and, crucially, who also had interacted on subreddits that do not have a geographical component. We found that the red and green lines overlap (Fig. 4), and that non-geographically salient users still preferentially interacted with others in closer geographical proximity (green line), suggesting that the observed decay with distance was not dependent on our localization procedure. That is to say, users from Seattle are not only more likely to interact with each other in r/seattle but also in other, non-location subreddits. That is not entirely surprising as online interactions have been shown to be bounded by geography, not least because social networks are based on real-world friends/contacts (as an example, we applied the same $Connectivity_d$ formula to a publicly available Facebook graph, and, in Supplementary Material, we observe that interactions on Facebook are even more geographically bounded than those on Reddit). Yet, in the case of Reddit, this result is remarkable because the platform is an anonymous forum where both a user’s identity and physical location are hidden from other users. Such Reddit’s anonymity lifts social pressure, and so geographically-bounded information spreading is more likely to stem, not from homophily at the circle-of-friends level (as in other social networks), but from people having like-minded individuals in their locations (i.e., states).

**Figure 5:**
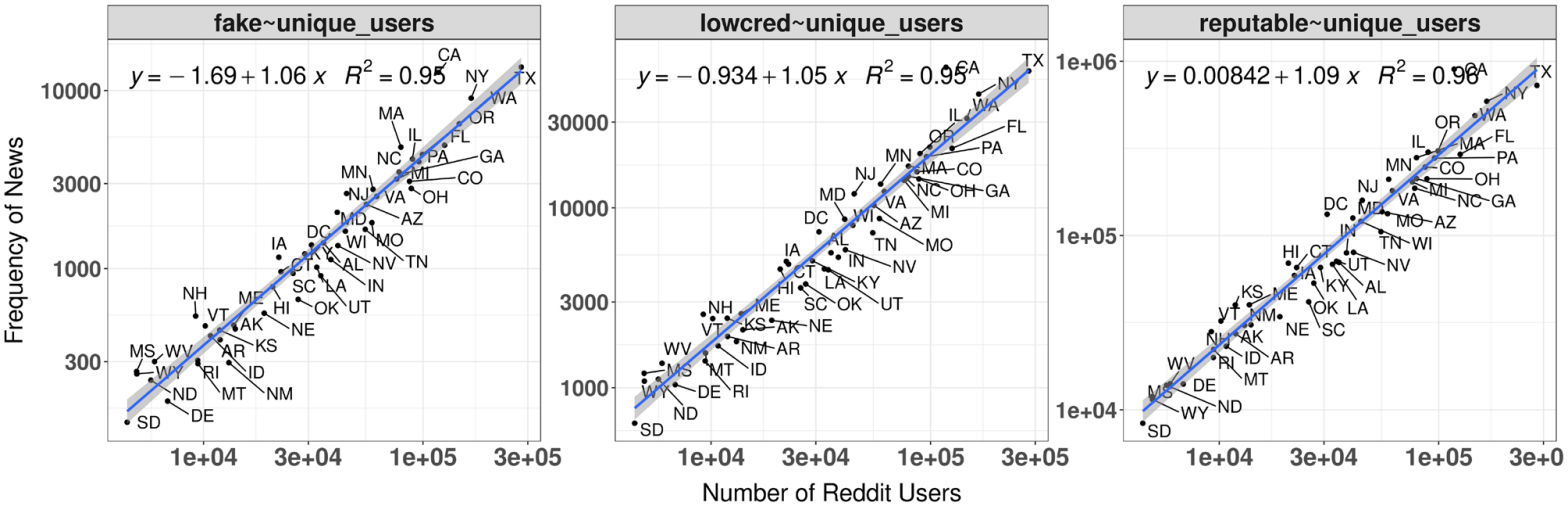
The scaling of news circulation. The *x*-axis is the total number of Reddit users from a state, and the *y*-axis denotes the number of posts containing each of the three types of news. We observed that the circulation of news approximates a supply and demand system (i.e., $\beta \approx 1.0$).

### The scaling laws of news circulation

Given that interactions are geographically bounded, it was reasonable to hypothesize that a state’s news circulation is best explained by the state’s variables rather than platform-specific variables. As previously mentioned, based on the scaling laws literature, one of these state variables is the number of users. We indeed found evidence that the number of Reddit users in a state is an important predictor of news circulation. It alone explained 95% ($R\approx 0.95$) of the variance: 1 unit log scale gain in number of users is approximately correlated with exactly 1 unit log scale gain in news circulation ($\beta \approx 1$) for all three types of news (Fig. 5), suggesting that news circulation on Reddit works as a supply-and-demand system.

### The role of the big sort

**Figure 6:**
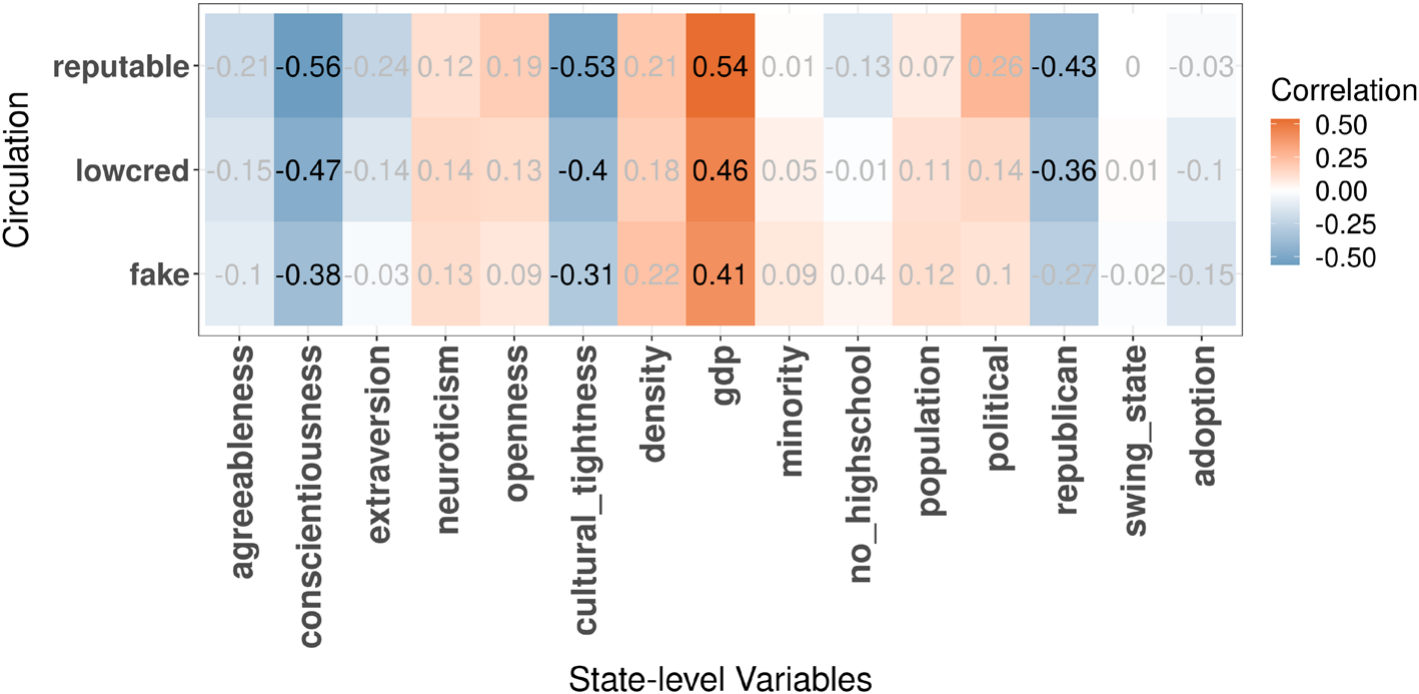
Correlation between circulation and each independent variable. Statistically insignificant correlations (*p*-value$\ge 0.05$) are grayed out. The matrix was created using version 0.92 of the following R package https://cran.r-project.org/web/packages/corrplot.

**Table 3:** Residual circulation regression results.

	Dependent variable: circulation					
	Reputable (personality and culture)	Reputable (complete)	Lowcred (personality and culture)	Lowcred (complete)	Fake (personality and culture)	Fake (complete)
	(1)	(2)	(3)	(4)	(5)	(6)
Agreeableness	0.022 (0.014)	0.022 (0.015)		0.038$$^{**}$$ (0.017)		0.033 (0.020)
Conscientiousness	− 0.052$$^{***}$$ (0.015)	− 0.053$$^{***}$$ (0.015)	− 0.040$$^{**}$$ (0.016)	− 0.054$$^{***}$$ (0.018)	− 0.044$$^{***}$$ (0.016)	− 0.040$$^{*}$$ (0.021)
Openness						−0.034 (0.023)
Cultural_tightness	− 0.038$$^{***}$$ (0.014)	− 0.025 (0.016)	− 0.025 (0.016)	− 0.027 (0.018)		− 0.036 (0.024)
No_highschool				0.032$$^{**}$$ (0.015)		0.054$$^{**}$$ (0.021)
Gdp		0.052$$^{**}$$ (0.019)		0.030$$^{*}$$ (0.017)		0.046$$^{**}$$ (0.020)
Density		− 0.027 (0.017)				
Political		− 0.023 (0.014)				
Constant	− 0.006 (0.012)	− 0.006 (0.011)	− 0.002 (0.014)	− 0.002 (0.013)	0.001 (0.016)	0.001 (0.015)
Observations	48	48	48	48	48	48
R$^{2}$	0.432	0.521	0.261	0.401	0.142	0.349
Adjusted R$^{2}$	0.393	0.451	0.228	0.329	0.123	0.254
Residual Std. Error	0.081 (df = 44)	0.077 (df = 41)	0.096 (df = 45)	0.089 (df = 42)	0.110 (df = 46)	0.102 (df = 41)
F Statistic	11.158$$^{***}$$ (df = 3; 44)	7.438$$^{***}$$ (df = 6; 41)	7.951$$^{***}$$ (df = 2; 45)	5.616$$^{***}$$ (df = 5; 42)	7.608$$^{***}$$ (df = 1; 46)	3.665$$^{***}$$ (df = 6; 41)

$$^{*}$$p < 0.1; $$^{**}$$p < 0.05; $$^{***}$$p < 0.01.

The *personality and culture* models (1)(3)(5) only used personality and cultural explanatory variables. The *complete* models (2)(4)(6) used all explanatory variables. For all models, stepAIC selected the most predictive subset of predictors. The predictors not shown are those that were not selected by StepAIC to be part of the optimal model.

To explore why news circulation might deviate from the supply-and-demand model at times, we studied the associations between the news circulation residual metric ${Residual}\ Circulation(s, i)$ and state-level attributes. Cultural tightness and conscientiousness had the highest correlation (absolute value) with circulation across all news types (Fig. 6), not least because the two variables are correlated with each other ($r[cultural\_tightness, conscientiousness]= 0.47, p < 0.05$ in Fig. 2). This translates into saying that conscientious states with restrictive social norms circulated fewer news items than what was expected by their Reddit adoption. The association was even more prominent for *reputable* news. For example, the correlation between *cultural tightness* and *Circulation* for *fake* news was $-\,\,0.31$; the correlation was $-\,\, 0.53$ for *reputable* news. In other words, users from states ranked high in *conscientiousness* were posting fewer reputable and fake news items than what was expected from their numbers of Reddit users. Next, focusing on political variables, we found that the presence of *republican* voters was noticeably negatively correlated with circulation of reputable and low-credibility news but not of fake news (in Fig. 6, *r*[*circulation*, *republican*] is negative for *reputable* and *lowcred*, but becomes insignificant for *fake*). That result is in line with prior studies showing that the majority of misinformation is conservative-leaning^5,75^. Also, that result has an additional explanation: states that are slightly more likely to use Reddit are democratic ones ($r[adoption, republican]= - 0.23, p >= 0.05$ in Figure 2), as further detailed in Supplementary Material. Surprisingly, we also saw that swing states with competitive political races were not more likely to circulate significantly more news. Finally, focusing on socioeconomic factors, we observed that wealthy states had higher circulation, irrespective of news types.

Next, we focused on the combined effects of state-level attributes by studying each news type separately. For each, we ran 3 partial regression models (personality and culture, socio-economic, and political) plus one combined model. Each of the models (3 partial + 1 complete) was then fitted using *stepAIC*, a method that statistically identifies the best combination of independent variables that lead to the best fit^76^. AIC estimates the model’s prediction error (the lower the value, the better the fit of the model), and its values should not be taken at face value but are best interpreted in a comparative fashion, allowing for model comparison. We ascertained that there was no multicollinearity among our predictors by computing their Variance Inflation Factor (VIF) scores^77^, and finding them to be $\le 2.5$ (scores larger than 5 indicate multicollinearity). Since we were interested in which variables (personality and culture *vs.* socio-economic *vs.* political) best explained news circulation, we report both the complete model and the partial model based on personality plus culture here (Table 3), and report the two other partial models in Supplementary Material. The StepAIC method chooses the best combination of predictors for a given dependent variable. Hence, the variables not shown in Table 3 are those that were not selected by StepAIC as predictors of the optimal model. We found that the complete models were able to explain a considerable fraction of variances in circulation residual (adjusted $R^2\approx \{0.25, 0.45\}$ in Table 3). The obtained adjusted R$^2$ values allowed us to compare the importance of different factors. That was possible because these values, despite being moderate, were akin or above the values found in similar studies, such as the adjusted R$^2$ of 0.08–0.51 when predicting crime rates from state outcomes^78^, or the correlations of 0.10–0.65 between upward income mobility and Facebook data-derived social capital indices^79^. Further, the variable *conscientiousness* was a significant indicator for lower-than-expected circulation for all types of news for all models; whereas *gdp* was significantly correlated with higher-than-expected circulation. More interestingly, we also saw that, for the personality and culture partial models, the adjusted $R^2 \approx \{0.12, 0.39\}$. In other words, the $R^2$ differences between the personality and culture models and the complete models were small. As an example, the adjusted $R^2$ for the full model for reputable news was 0.45, whereas the adjusted $R^2$ for the personality and culture model was 0.39 (a difference of only 0.06). In fact, including personality and cultural variables improved the full models’ adjusted $R^2$ from 0.10 to 0.20 (see Supplementary Material). Additionally, we also saw that personality and culture models had higher adjusted $R^2$ values than, as Supplementary Material shows, models that exclusively used socioeconomic conditions (adjusted $R^2\approx \{0.15, 0.29\}$) or political characteristics (adjusted $R^2\approx \{0.06, 0.21\}$). As a robustness check, we also reran our analysis using normalized circulation volume. Specifically, we redefined *Circulation*(*s*, *i*) as the average number of comments containing URLs to news type *s* posted by Reddit users from state *i*. We then reran Eq. (1). The main findings detailed in Supplementary Material did not change: personality and cultural factors still remained strong indicators of circulation.

Finally, by comparing the values of the beta coefficients for different news types in Table 3, we observed that circulation of any news types was facilitated in states that: are wealthier (*gdp* has positive *beta*’s in Table 3), have residents who are less diligent in terms of personality (*conscientiousness* has negative *beta*’s), and are characterized by loose cultures which understate the importance of adherence to norms ($cultural\_tightness$ has negative *beta*’s). That holds for all types of news. We then focused on the circulation of misinformation specifically, and observed that was taking place once these three factors were combined with a fourth one: low education levels ($no\_highschool$ has a positive *beta* in the complete fake news model in Table 3).

## Discussion

Our first finding is that platform-facilitated news diffusion within Reddit is limited. Specifically, we observed that geographical diffusion is rare (for example, only 6% of news had reached 5 or more states), as is diffusion from person to person (for example, 75% of all reputable news articles were only posted by a single user). This is in contrast with previous work, which found that other types of social networks (e.g., Facebook and Twitter) work as a “Hype Machine”^16^.Our contrasting results likely stem from the moderation mechanism that Reddit employs to avoid the reposting of the same content, and the posting of highly emotionally-charged content. Namely, volunteer moderators run each subreddit, settle disputes, and decide who may or may not participate. They also levy rules on what is appropriate, and what content will stay online as is, be edited, or deleted. A recent study^80^ estimated that in 2020, the volunteer moderators’ labour, if they were commercial moderators, would cost Reddit 2.8 per cent of the company’s total revenue in 2019. Importantly, these volunteer moderators have a close connection with their respective communities and in-depth knowledge about community dynamics, which commercial moderators might not be able to replace.

Our second finding is that Reddit users who are geographically close are more likely to interact, even if we were to remove the interactions that took place in city- or state-related subreddits. This finding is in line with previous literature, which showed that the probability of interaction in any social network exponentially falls with physical distance^72,81,82^.

Our third finding is that news circulation on Reddit works as a supply-and-demand system. We indeed found the scaling exponent of $\beta$ to be exactly 1 (linear) instead of being above 1 (superlinear). This is an interesting finding as linear scaling is associated with elements that require individual maintenance (e.g., water pipes), while superlinear scaling is associated with the “creation of information, wealth and resources”^69^, which could have included the circulation of news online. The unitary scaling points to a novel finding, in that, online news circulation is not amplified on Reddit (as per the Hype Machine hypothesis^16,83^) but simply meets the demand.

Our fourth and last finding is that deviations from the supply-and-demand model are mostly explained by geographical factors. This is a new finding since the geographical side of online news has received little attention. Furthermore, we found that these factors include state-level personality and cultural factors rather than, as it could have been hypothesized from previous studies^12,84,85^, socio-economic conditions or political characteristics.

Our work has one main ramification for research focused on “why” do people share news and, relatedly, on “how” to curtail the spread of misinformation. This has to do with the stability of personality and culture. Adding to that the fact that we geographically cluster with similar ones because that increases life satisfaction, the potential for algorithms to influence the way we share information (including combating misinformation) is limited, at least for Reddit. Hence, we would be better off combating the production of misinformation altogether rather than changing its circulation once it has been created. More specifically, personality and culture are ingrained parts of every individual; they generally remain stable for people who have reached adulthood^86^. Moreover, past research showed that individuals are likely drawn to regions that match their personality and cultural norms as this matching increases their overall life satisfaction^30^. In fact, prior longitudinal analysis on state-wide personality traits showed that states’ big-5 personality ranks remained unchanged in the last 20 years^34^. Given such level of “stability” and clustering, these traits are likely to affect news diffusion beyond the effects of the platform algorithms, and, hence, make combating misinformation more difficult (for instance, it would be difficult to compel “unconscientious personalities” to be more conscientious^87^). Social media platforms’ recommendation and personalization algorithms had led to the formulation of homogeneous, tight-knit communities en mass. These communities had then facilitated the circulation of *(mis)information*. Thus, researchers had proposed various ways to regulate these algorithms, including increasing the diversity of perspectives and connections available to users. Yet, our results suggest that algorithmic amplification is not the main driver of news circulation, at least not in the case of Reddit. Rather, among the main drivers is geographic sorting that has been happening in the last 40 years. Given these considerations, we argue that a more productive way to combat misinformation is to reduce its production altogether. That is, we need to disincentivize the creation of fake and low-credibility news sites and news content before they can be shared by individuals and online communities. This can be done in several ways. For instance, many fake news sites are driven by ad profit^19^. As such, ad firms and retailers can curtail misinformation by blacklisting known fake and low-credibility news sites, and recent research suggested that, in so doing, major ad firms would not suffer any significant loss of revenues^88^. Similarly, lawmakers can also pass regulations such as criminalizing false stories (e.g., laws against defamation in the offline world already exist) with the potential to ignite communal tension^89^.

There are five main limitations to our work. First, our work was exclusively focused on news circulation, and, as such, we did not address its actual consumption (e.g., we cannot determine the number of users who actually read and believed the content from the posted news URLs, but could only determine the number of those who were potentially exposed).

Second, our project solely relied on Reddit data, and we do not know whether our results generalize to other platforms. Reddit is an anonymous platform without the concept of ‘friends’, unlike many other social networks. As such, Reddit users are less likely to form echo chambers. Hence, geographically-bounded information spreading is more likely to stem, not from belonging to the same circles of friends (as in other social networks), but from sharing similar interests. We cannot be sure that Reddit does not have a mechanism under the hood that encourages geographically-bounded interactions; however, since users are free to create and join subreddits of interest, that does not seem likely. Moreover, in Supplementary Material, we showed that interactions on Facebook are even more geographically localized that those on Reddit, suggesting that geographic segregation might play an even stronger role on Facebook.

Third, we approximated a user’s geolocation at the state level because that was the granularity allowed by Reddit. The probabilistic procedure with which Reddit users were geolocated effectively works at state level (e.g., correlation of .89 to .95 of the number of users with census population)^38,90^. However, it limits the ability to disentangle news circulation between urban and rural areas. A state’s personality and culture, socioeconomic, and political attributes can vary significantly from one sub-region to another, including between rural and urban areas in the same state^91^. Future work might attempt to perform a similar geolocation analysis at a finer granularity (e.g., at city level) on platforms that allow for it.

Fourth, we labeled articles to represent misinformation based on their publishers and not on their content. This approach is widely used in misinformation studies^39^, in part because it is hard to label every single article, and do so accurately, as this would require extensive investigation of what is true and what is false in each single event being covered. (For the same reasons, selection bias may arise when using article-level labels, as fact-checkers are time and resource constrained and might select only certain types of news that they consider significant and newsworthy.) A recent study showed that corporate fake news is negatively associated with a company’s contemporaneous abnormal return and positively associated with contemporaneous abnormal turnover, and this result was independent of whether fakeness was defined using publisher-level or article-level credibility scores^92^. We also performed a Groundtruth Labels Robustness Check (in Supplemental Information) against trustworthiness scores provided by professional fact-checkers. We found following trustworthiness scores for each of our categories: reputable (0.66), low-credibility (0.1) and fake news sites (0.02), indicating that our publisher-level credibility scores align well with the article-level ratings by professional fact-checkers.

Fifth, our data did not contain comments that were deleted prior to being collected by pushshift.io. As such, we could not examine whether those deleted comments contained news URLs. In particular, comments that were removed by Automoderator (bots) were unavailable to us, as these comments were removed as soon as they were posted. Nevertheless, the Reddit dataset from  pushshift.io remains one of the most comprehensive datasets available^37^. Furthermore, reputable news is unlikely to be removed by moderators, and our observations for true news still showed the prominent role of regional personality and culture, speaking to the robustness of our findings.

## Supplementary Information

Supplementary Information.

## Data Availability

We made publicly available the following data: (1) *geolocated Reddit users* (3M identifiers of users who were located in one of the 50 U.S. states), (2) *news comments from those Reddit users* (8.23M comments containing news links), (3) *names of news sites* (news sites and their corresponding categories: fake, lowcred, and reputable), and (4) *US state-level attributes* (personality and cultural, socio-economic, and political). A detailed description of how we created the data and how to retrieve it is available at the following link https://doi.org/10.6084/m9.figshare.20223867.v1.
