# Supplementary material for: The role of the big geographic sort in online news circulation among U.S. Reddit users
Source: Sci Rep. 2023 Apr 25;13:6711. doi: 10.1038/s41598-023-33247-3 (PMC10126553; doi:10.1038/s41598-023-33247-3)
Supplement: Supplementary file 1 — Supplementary Information. [file 41598_2023_33247_MOESM1_ESM.pdf]

Supplementary Material — The role of the Big Geographic Sort in Online News Circulation among U.S. Reddit Users

immediate

Data

Reddit User Representativeness

In this section, we included additional analysis focused on the representativeness of Reddit users with respect to party affiliation. Results are summarized on Figure S1a and Figure S1b. For Figure S1a, the x-axis is calculated as the percentage of population leaning Republican minus percentage of population leaning Democrat. The y-axis is the Reddit adoption rate which was calculated as the number of Reddit users from a given state divided by the state's population. As shown, democratic-leaning states had a significantly higher adoption rate than Republican-leaning states. Next, we also computed the average number of Reddit comments by users from each state and plotted the result against each state's political affiliation. As shown in Figure S1b, users from Democratic-leaning states contributed more comments on average than Republican-leaning states. Results suggest that Democratic-leaning states are over-represented compared to Republican-leaning states due to having more users present on Reddit and also the users being more active.

Geotagged Users VS. Non-geotagged Users

We first sampled approximately 3 million users that were not matched to one of the 50 U.S. states. We then compared the Reddit activity levels of these non-geotagged users to the set of geotagged users (those that were assigned to one of the U.S. states). We observed that the numbers of total Reddit comments posted by users of the two groups are comparable. Specifically, an average geotagged user posted a total of 88 comments and an average non-geotagged user posted 65 comments. However, we also saw that geotagged users had higher numbers of news comments. For instance, 1.7% of all geotagged users had posted at least 1 comment containing fake news URLs, whereas only 0.6% of non-geotagged users did. Similarly, 28.2% of all geotagged users had posted at least 1 comment containing reputable news URLs, but only 19.2% of non-geotagged users did. Results are shown in Figure S2. The difference here can be explained by non-geotagged users being less likely to be from the U.S., and thus less invested in the ongoing of the U.S.

Groundtruth Labels Robustness Check

Here, we compared our news categorization (*fake, lowcred* and *reputable*) to the trustworthiness scores provided by related work[45]. Specifically, Pennycook et al.[45] provided a list of 60 news sites with trustworthiness ratings ($0-100$ where 100 is completely trustworthy) from professional fact-checkers. As shown in Figure S3, of the 60 news sites, those labeled as reputable in our paper had the highest trustworthiness scores (average score was 0.66), followed by those labeled as low-credibility (average score was 0.1) and then fake news sites (average score was 0.02). Results here indicate that our classification labeling is aligned with the ratings by professional fact-checkers.

Results

Geographical Segregation on Facebook

We compared the extent of Reddit users' geographical segregation to that of Facebook's. We use the Social Connectivity Index (SCI) of states from Bailey et al.[?]. The SCI between states i and j is calculated as the probability that a randomly selected pair of users from i and j respectively are Facebook friends (the original work[?] had then scaled SCI scores for all geographical regions in the world to the range of 1 to 1 billion). Here, we determined the median SCI for pairs of states that are approximately d distance apart where $d = \{0km, 100km, 200km...\}$ (distance between 2 states were measured using their geographic centers). As shown in Figure S4, we observed that SCI dropped sharply when $i \neq j$. In other words, most Facebook friends are from the same state. Additionally, the geographical segregation is more prominent on Facebook compared to Reddit. For instance, the SCI index dropped by a factor of 208 from $d = 0$ to $d = 100km$. In comparison *Connectivity* on Reddit only dropped by a factor of 20.

The Big Sort

Additional analyses in this section demonstrated that our initial findings are robust.

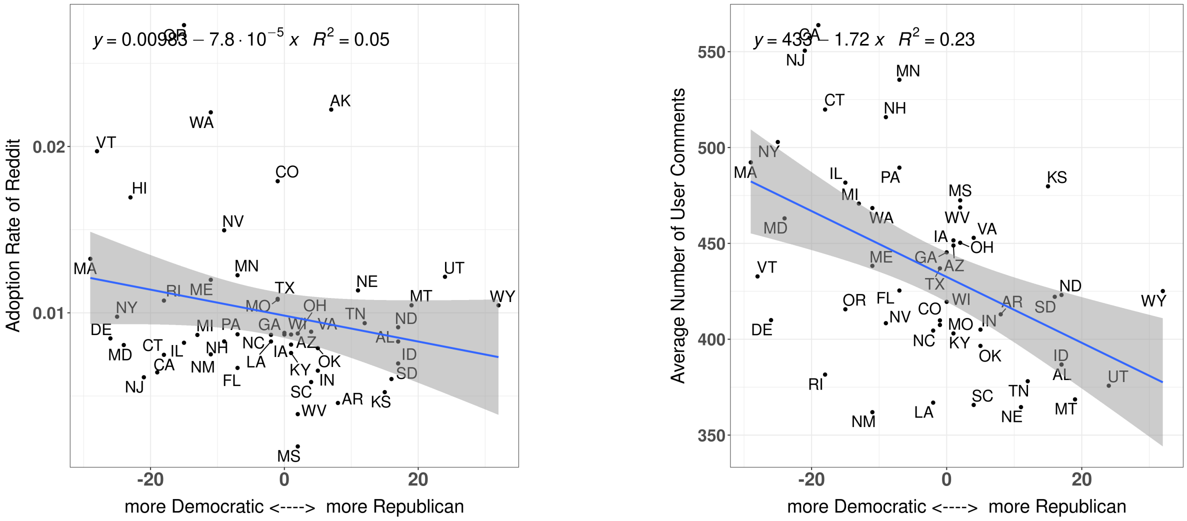

(a) Correlation between state's political leaning and its Reddit adoption rate.

(b) Correlation between state's political leaning and its Reddit activity level.

Figure S1. Reddit Data Representativeness

Table S1. Regression Results for Circulation Using Only Socioeconomic Attributes. We saw that states with a higher average income had higher-than-expected circulation for all news types. More importantly, the variance explained by socioeconomic conditions was comparable or slightly lower than the variance explained by personality and cultural factors (see Table 3).

	Dependent variable: circulation		
	reputable	lowcred	fake
	(1)	(2)	(3)
no_highschool		0.035 (0.022)	
gdp	0.071*** (0.017)	0.066*** (0.017)	0.049*** (0.016)
density	−0.024 (0.017)		
minority		−0.034 (0.022)	
Constant	−0.006 (0.013)	−0.002 (0.014)	0.001 (0.016)
Observations	48	48	48
R^2	0.319	0.256	0.170
Adjusted R^2	0.289	0.206	0.152
Residual Std. Error	0.087 (df = 45)	0.097 (df = 44)	0.108 (df = 46)
F Statistic	10.530*** (df = 2; 45)	5.053*** (df = 3; 44)	9.451*** (df = 1; 46)
Note:			*p<0.1; **p<0.05; ***p<0.01

Types of state-level attributes

We included separate regression models fitted with only socioeconomic variables (Table S1) or political variables (Table S2). We then compared the R^2 of these models with the models fitted with only personality and cultural variables (Table 3 in the main text). We observed that the models fitted with only personality and cultural variables had the highest R^2 than models fitted with only socioeconomic or political variables. Next, by comparing the full models (Table 3 in the main text) and models that included all variables except for personality and culture (Table S3), we also saw that adding personality and cultural variables increased the R^2 values by 0.1 to 0.2. Both analyses demonstrated that personality and cultural factors are strong indicators of circulation.

Circulation Fraction

In this section, we redefined $Circulation(s,i)$ as the average number of comments containing URLs to news type s posted by Reddit users from state i. We then reran Equation 1 from the main text. Results are summarized in Table S4. As shown,

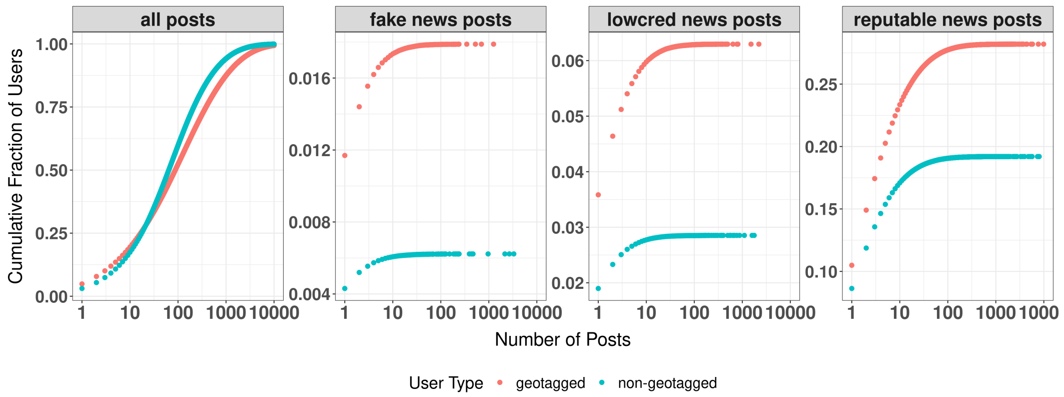

Figure S2. Comparing Geotagged and Non-geotagged Users. We saw that users are comparable in terms of overall comments. However, geotagged users posted more news comments. For instance, approximately 30% of all geotagged users had posted at least 1 comment containing URLs from reputable news sites. In comparison, only 20% of non-geotagged users did.

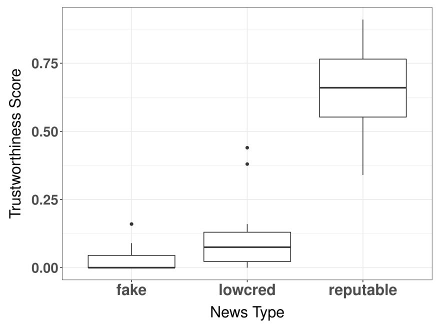

Figure S3. The distribution of trustworthiness scores for each news type.

Table S2. Regression Results for Circulation Using Only Political Attributes. We saw that Republican-leaning and politically disengaged states had lower-than-expected circulation. More importantly, we also observed that the variance explained by political attributes was much lower than the variance explained by personality and cultural factors (see Table 3 in the main text).

	Dependent variable: circulation		
	reputable	lowcred	fake
	(1)	(2)	(3)
republican	−0.043*** (0.013)	−0.040** (0.015)	−0.032* (0.017)
political	0.026* (0.013)		
Constant	−0.006 (0.013)	−0.002 (0.015)	0.001 (0.016)
Observations	48	48	48
R^2	0.246	0.131	0.075
Adjusted R^2	0.213	0.112	0.055
Residual Std. Error	0.092 (df = 45)	0.103 (df = 46)	0.114 (df = 46)
F Statistic	7.342*** (df = 2; 45)	6.939** (df = 1; 46)	3.754* (df = 1; 46)
Note:			*p<0.1; **p<0.05; ***p<0.01

personality and cultural variables were still strongly indicative of circulation for all news types. For instance, the reputable news circulation model that included only personality and cultural variables had an adjusted R^2 of 0.32. In comparison, the full model's R^2 was 0.57. Furthermore, we again saw that *conscientiousness* was significantly and negatively correlated with the circulation of all news types. Additionally, the coefficient for *cultural tightness* is negative although not significant.

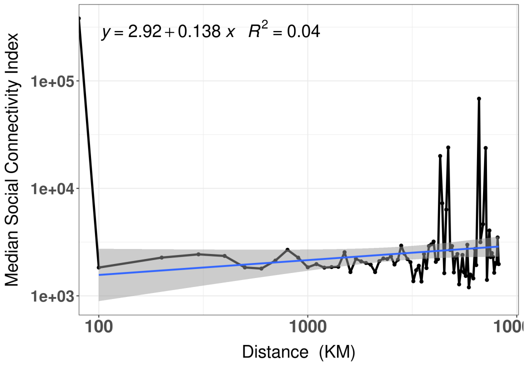

Figure S4. Facebook Social Connectivity Index. The x-axis denotes the geographical distance and the y-axis is the median social connectivity index between regions that are *x* distance apart**?**. We observed a similar, albeit much more extreme, pattern of geographical segregation.

Table S3. Regression Results for Circulation Using All Attributes Except for Personality and Culture. We saw that personality and cultural factors explained any additional 10% to 20% of the variance. As an example, the full model for reputable news using all variables had adjusted $R^2 = 0.45$. Here, we saw that the same model excluding personality and culture only had adjusted $R^2 = 0.338$ (a difference of approximately 0.11).

	Dependent variable: circulation		
	reputable	lowcred	fake
	(1)	(2)	(3)
no_highschool		0.033 (0.022)	
gdp	0.062*** (0.017)	0.072*** (0.022)	0.049*** (0.016)
minority		−0.031 (0.022)	
density	−0.038** (0.017)	−0.033 (0.020)	
republican	−0.033** (0.016)	−0.025 (0.019)	
Constant	−0.006 (0.012)	−0.002 (0.014)	0.001 (0.016)
Observations	48	48	48
R^2	0.380	0.313	0.170
Adjusted R^2	0.338	0.231	0.152
Residual Std. Error	0.084 (df = 44)	0.096 (df = 42)	0.108 (df = 46)
F Statistic	9.003*** (df = 3; 44)	3.831*** (df = 5; 42)	9.451*** (df = 1; 46)
Note:			*p<0.1; **p<0.05; ***p<0.01

Satirical News

Circulation analyses for satirical news are summarized in Table S5. We included 3 partial models (1)(2)(3), each including only one category of state-level attributes, plus a 4th full model which contained all explanatory variables. Note that all models were fitted with stepAIC. We again saw that the difference in R^2 was small for the model that included only the personality and cultural variables and the full model (a difference of $0.372 - 0.342 = 0.03$). Additionally, *cultural tightness* is significantly and negatively correlated with circulation of satirical news. Despite the coefficient for *conscientiousness* being not significant, we noted its directionality remained negative ($\beta < 0$).

Table S4. Regression Results for Circulation Fraction. Here, the dependent variable was calculated as the average number of comments posted by users from a given state for each news type. We observed comparable results: personality and culture were strong indicators of circulation.

	Dependent variable: circulation fraction					
	reputable mod (personality and culture)	reputable mod (complete)	lowcred mod (personality and culture)	lowcred mod (complete)	fake mod (personality and culture)	fake mod (complete)
	(1)	(2)	(3)	(4)	(5)	(6)
extraversion	0.208* (0.123)		0.013 (0.009)		0.004* (0.002)	
agreeableness	0.283* (0.143)	0.239** (0.118)	0.019* (0.010)	0.021** (0.009)	0.004* (0.002)	0.005** (0.002)
conscientiousness	−0.398** (0.153)	−0.349*** (0.120)	−0.029** (0.011)	−0.024** (0.009)	−0.006** (0.003)	−0.004* (0.002)
openness	0.329** (0.133)		0.018* (0.010)		0.004* (0.002)	−0.003 (0.002)
cultural_tightness	−0.306** (0.136)	−0.344*** (0.123)	−0.017 (0.010)	−0.019** (0.009)	−0.003 (0.002)	−0.006** (0.002)
no_highschool		0.294** (0.122)		0.032*** (0.008)		0.009*** (0.002)
population		0.267** (0.122)				
gdp		0.373** (0.160)		0.032*** (0.011)		0.007*** (0.002)
density		−0.229* (0.124)		−0.016 (0.010)		
Constant	2.676*** (0.107)	2.676*** (0.085)	0.194*** (0.008)	0.194*** (0.007)	0.042*** (0.002)	0.042*** (0.001)
Observations	48	48	48	48	48	48
R^2	0.396	0.638	0.311	0.523	0.275	0.496
Adjusted R^2	0.324	0.574	0.229	0.453	0.189	0.423
Residual Std. Error	0.740 (df = 42)	0.587 (df = 40)	0.054 (df = 42)	0.045 (df = 41)	0.012 (df = 42)	0.010 (df = 41)
F Statistic	5.515*** (df = 5; 42)	10.056*** (df = 7; 40)	3.797*** (df = 5; 42)	7.478*** (df = 6; 41)	3.187** (df = 5; 42)	6.735*** (df = 6; 41)
Note:						*p<0.1; **p<0.05; ***p<0.01

Table S5. Circulation of Satirical News. We again saw that that personality and cultural factors (specifically, *conscientiousness* and *cultural tightness*) alone explained a considerable portion of the variance.

	Dependent variable: circulation			
	personality and culture	socioeconomic	political	complete
	(1)	(2)	(3)	(4)
conscientiousness	−0.047** (0.019)			
agreeableness				0.033 (0.021)
neuroticism				0.048** (0.019)
cultural_tightness	−0.054*** (0.019)			−0.050** (0.021)
gdp		0.091*** (0.017)		0.067*** (0.021)
minority		−0.042** (0.017)		
republican			−0.051** (0.019)	
political			0.035* (0.019)	
Constant	−0.008 (0.017)	−0.008 (0.016)	−0.008 (0.019)	−0.008 (0.016)
Observations	48	48	48	48
R^2	0.370	0.398	0.193	0.455
Adjusted R^2	0.342	0.372	0.157	0.405
Residual Std. Error	0.116 (df = 45)	0.113 (df = 45)	0.131 (df = 45)	0.110 (df = 43)
F Statistic	13.229*** (df = 2; 45)	14.897*** (df = 2; 45)	5.369*** (df = 2; 45)	8.984*** (df = 4; 43)
Note:				*p<0.1; **p<0.05; ***p<0.01

References

1. Kümpel, A. S., Karnowski, V. & Keyling, T. News sharing in social media: A review of current research on news sharing users, content, and networks. *Soc. media+ society* **1**, 2056305115610141 (2015).

2. Forgas, J. P. & Baumeister, R. *The Social Psychology of Gullibility: Conspiracy Theories, Fake News and Irrational Beliefs* (Routledge, 2019).

3. Burbach, L., Halbach, P., Ziefle, M. & Calero Valdez, A. Who shares fake news in online social networks? In *Proceedings of the 27th ACM Conference on User Modeling, Adaptation and Personalization*, 234–242 (2019).

4. Buchanan, T. & Benson, V. Spreading disinformation on facebook: Do trust in message source, risk propensity, or personality affect the organic reach of "fake news"? *Soc. Media+ Soc.* **5**, 2056305119888654 (2019).

5. Grinberg, N., Joseph, K., Friedland, L., Swire-Thompson, B. & Lazer, D. Fake news on twitter during the 2016 us presidential election. *Science* **363**, 374–378 (2019).

6. Balestrucci, A. & De Nicola, R. Credulous users and fake news: a real case study on the propagation in twitter. In *2020 IEEE Conference on Evolving and Adaptive Intelligent Systems (EAIS)*, 1–8 (IEEE, 2020).

7. Kim, K., Baek, Y. M. & Kim, N. Online news diffusion dynamics and public opinion formation: a case study of the controversy over judges' personal opinion expression on sns in korea. *The Soc. Sci. J.* **52**, 205–216 (2015).

8. Xiao, X. & Su, Y. Wired to seek, comment and share? examining the relationship between personality, news consumption and misinformation engagement. *Online Inf. Rev.* (2022).

9. MIAN, L. S. The effects of negative emotions and personality on news sharing behaviour. (2020).

10. Ling, R. Confirmation bias in the era of mobile news consumption: the social and psychological dimensions. *Digit. Journalism* **8**, 596–604 (2020).

11. Amazeen, M. A., Vargo, C. J. & Hopp, T. Reinforcing attitudes in a gatewatching news era: Individual-level antecedents to sharing fact-checks on social media. *Commun. Monogr.* **86**, 112–132 (2019).

12. Kalogeropoulos, A., Negredo, S., Picone, I. & Nielsen, R. K. Who shares and comments on news?: A cross-national comparative analysis of online and social media participation. *Soc. media+ society* **3**, 2056305117735754 (2017).

13. Ihm, J. & Kim, E.-m. The hidden side of news diffusion: Understanding online news sharing as an interpersonal behavior. *New Media & Soc.* **20**, 4346–4365 (2018).

14. An, J., Quercia, D. & Crowcroft, J. Partisan sharing: Facebook evidence and societal consequences. In *Proceedings of the second ACM conference on Online social networks*, 13–24 (2014).

15. Scherer, L. D. *et al.* Who is susceptible to online health misinformation? a test of four psychosocial hypotheses. *Heal. Psychol.* (2021).

16. Aral, S. *The Hype Machine: How Social Media Disrupts Our Elections, Our Economy, and Our Health–and How We Must Adapt* (Currency, 2020).

17. Pariser, E. *The filter bubble: How the new personalized web is changing what we read and how we think* (Penguin, 2011).

18. Jamieson, K. H. & Cappella, J. N. *Echo chamber: Rush Limbaugh and the conservative media establishment* (Oxford University Press, 2008).

19. Bakir, V. & McStay, A. Fake news and the economy of emotions: Problems, causes, solutions. *Digit. journalism* **6**, 154–175 (2018).

20. Rathje, S., Van Bavel, J. J. & van der Linden, S. Out-group animosity drives engagement on social media. *Proc. Natl. Acad. Sci.* **118** (2021).

21. Vosoughi, S., Roy, D. & Aral, S. The spread of true and false news online. *Science* **359**, 1146–1151, DOI: 10.1126/science.aap9559 (2018).

22. Leskovec, J., Backstrom, L. & Kleinberg, J. Meme-tracking and the dynamics of the news cycle. In *Proceedings of the 15th ACM SIGKDD international conference on Knowledge discovery and data mining*, 497–506 (2009).

23. Yang, J. & Leskovec, J. Modeling information diffusion in implicit networks. In *2010 IEEE International Conference on Data Mining*, 599–608 (IEEE, 2010).

24. Myers, S. A. & Leskovec, J. Clash of the contagions: Cooperation and competition in information diffusion. In *2012 IEEE 12th international conference on data mining*, 539–548 (IEEE, 2012).

25. Wang, X., Lan, Y. & Xiao, J. Anomalous structure and dynamics in news diffusion among heterogeneous individuals. *Nat. Hum. Behav.* **3**, 709–718 (2019).

26. Gravino, P., Prevedello, G., Galletti, M. & Loreto, V. The supply and demand of news during covid-19 and assessment of questionable sources production. *Nat. Hum. Behav.* 1–10 (2022).

27. Bishop, B. *The big sort: Why the clustering of like-minded America is tearing us apart* (Houghton Mifflin Harcourt, 2009).

28. Glass, J. & Levchak, P. Red states, blue states, and divorce: Understanding the impact of conservative protestantism on regional variation in divorce rates. *Am. J. Sociol.* **119**, 1002–1046 (2014).

29. Monson, R. A. & Mertens, J. B. All in the family: Red states, blue states, and postmodern family patterns, 2000 and 2004. *The Sociol. Q.* **52**, 244–267 (2011).

30. Jokela, M., Bleidorn, W., Lamb, M. E., Gosling, S. D. & Rentfrow, P. J. Geographically varying associations between personality and life satisfaction in the london metropolitan area. *Proc. Natl. Acad. Sci.* **112**, 725–730 (2015).

31. Scala, D. J. & Johnson, K. M. Political polarization along the rural-urban continuum? the geography of the presidential vote, 2000–2016. *The ANNALS Am. Acad. Polit. Soc. Sci.* **672**, 162–184 (2017).

32. Rentfrow, P. J., Jost, J. T., Gosling, S. D. & Potter, J. Statewide differences in personality predict voting patterns in 1996–2004 us presidential elections. *Soc. psychological bases ideology system justification* **1**, 314–349 (2009).

33. Rentfrow, P. J. *et al.* Divided we stand: Three psychological regions of the united states and their political, economic, social, and health correlates. *J. personality social psychology* **105**, 996 (2013).

34. Elleman, L. G., Condon, D. M., Russin, S. E. & Revelle, W. The personality of us states: Stability from 1999 to 2015. *J. Res. Pers.* **72**, 64–72 (2018).

35. Mullainathan, S. & Shleifer, A. The market for news. *Am. economic review* **95**, 1031–1053 (2005).

36. Gentzkow, M. & Shapiro, J. M. What drives media slant? evidence from us daily newspapers. *Econometrica* **78**, 35–71 (2010).

37. Baumgartner, J., Zannettou, S., Keegan, B., Squire, M. & Blackburn, J. The pushshift reddit dataset. In *Proceedings of the international AAAI conference on web and social media*, vol. 14, 830–839 (2020).

38. Balsamo, D., Bajardi, P. & Panisson, A. Firsthand opiates abuse on social media: monitoring geospatial patterns of interest through a digital cohort. In *The World Wide Web Conference*, 2572–2579 (2019).

39. Bozarth, L., Saraf, A. & Budak, C. Higher ground? how groundtruth labeling impacts our understanding of fake news about the 2016 us presidential nominees. In *Proceedings of the International AAAI Conference on Web and Social Media*, vol. 14, 48–59 (2020).

40. Vargo, C. J., Guo, L. & Amazeen, M. A. The agenda-setting power of fake news. *new media & society* **20**, 2028–2049 (2018).

41. Zimdars, M. My "fake news list" went viral. but made-up stories are only part of the problem. *The Wash. Post* (2016).

42. Politifact staff. Politifact guide to fake news websites and what they peddle (2018).

43. Couts, A. & Wyrich, A. Here are all the 'fake news' sites to watch out for on facebook (2016).

44. Allcott, H., Gentzkow, M. & Yu, C. Trends in the diffusion of misinformation on social media. *arXiv preprint arXiv:1809.05901* (2018).

45. Pennycook, G. & Rand, D. G. Fighting misinformation on social media using crowdsourced judgments of news source quality. *Proc. Natl. Acad. Sci.* **116**, 2521–2526 (2019).

46. Khan, M. L. & Idris, I. K. Recognise misinformation and verify before sharing: a reasoned action and information literacy perspective. *Behav. & Inf. Technol.* **38**, 1194–1212 (2019).

47. Bonney, K. M. Fake news with real consequences: the effect of cultural identity on the perception of science. *The Am. Biol. Teach.* **80**, 686–688 (2018).

48. Islam, A. N., Laato, S., Talukder, S. & Sutinen, E. Misinformation sharing and social media fatigue during covid-19: An affordance and cognitive load perspective. *Technol. Forecast. Soc. Chang.* **159**, 120201 (2020).

49. Calvillo, D. P., Garcia, R. J., Bertrand, K. & Mayers, T. A. Personality factors and self-reported political news consumption predict susceptibility to political fake news. *Pers. Individ. Differ.* **174**, 110666 (2021).

50. Soto, C. J. & John, O. P. The next big five inventory (bfi-2): Developing and assessing a hierarchical model with 15 facets to enhance bandwidth, fidelity, and predictive power. *J. personality social psychology* **113**, 117 (2017).

51. John, O. P., Srivastava, S. *et al.* The big-five trait taxonomy: History, measurement, and theoretical perspectives. (1999).

52. Rentfrow, P. J. Statewide differences in personality: toward a psychological geography of the united states. *Am. Psychol.* **65**, 548 (2010).

53. Deng, S., Lin, Y., Liu, Y., Chen, X. & Li, H. How do personality traits shape information-sharing behaviour in social media? exploring the mediating effect of generalized trust. *Inf. research: an international electronic journal* **22**, n3 (2017).

54. Matzler, K., Renzl, B., Müller, J., Herting, S. & Mooradian, T. A. Personality traits and knowledge sharing. *J. economic psychology* **29**, 301–313 (2008).

55. Gou, L., Zhou, M. X. & Yang, H. Knowme and shareme: understanding automatically discovered personality traits from social media and user sharing preferences. In *Proceedings of the SIGCHI conference on human factors in computing systems*, 955–964 (2014).

56. Witkin, H. A. & Berry, J. W. Psychological differentiation in cross-cultural perspective. *ETS Res. Bull. Ser.* **1975**, i–100 (1975).

57. Li, R., Gordon, S. & Gelfand, M. J. Tightness–looseness: A new framework to understand consumer behavior. *J. Consumer Psychol.* **27**, 377–391 (2017).

58. Babič, K., Černe, M., Škerlavaj, M. & Zhang, P. The interplay among prosocial motivation, cultural tightness, and uncertainty avoidance in predicting knowledge hiding. *Econ. Bus. Rev.* **20**, 395–422 (2018).

59. Harrington, J. R. & Gelfand, M. J. Tightness–looseness across the 50 united states. *Proc. Natl. Acad. Sci.* **111**, 7990–7995 (2014).

60. Deckert, C. & Schomaker, R. M. Cultural tightness–looseness and national innovativeness: impacts of tolerance and diversity of opinion. *J. Innov. Entrepreneurship* **11**, 1–19 (2022).

61. Mattison Thompson, F. & Brouthers, K. D. Digital consumer engagement: national cultural differences and cultural tightness. *J. Int. Mark.* **29**, 22–44 (2021).

62. McLeod, D. M. & Perse, E. M. Direct and indirect effects of socioeconomic status on public affairs knowledge. *Journalism Q.* **71**, 433–442 (1994).

63. Gil de Zúñiga, H., Jung, N. & Valenzuela, S. Social media use for news and individuals' social capital, civic engagement and political participation. *J. computer-mediated communication* **17**, 319–336 (2012).

64. Guess, A., Nagler, J. & Tucker, J. Less than you think: Prevalence and predictors of fake news dissemination on facebook. *Sci. advances* **5**, eaau4586 (2019).

65. Jones-Jang, S. M., Mortensen, T. & Liu, J. Does media literacy help identification of fake news? information literacy helps, but other literacies don't. *Am. Behav. Sci.* **65**, 371–388 (2021).

66. He, L., Yang, H., Xiong, X. & Lai, K. Online rumor transmission among younger and older adults. *Sage open* **9**, 2158244019876273 (2019).

67. McCann, A. Most and least politically engaged states (2020).

68. West, G. B. *Scale: the universal laws of growth, innovation, sustainability, and the pace of life in organisms, cities, economies, and companies* (Penguin, 2017).

69. Bettencourt, L. M., Lobo, J., Helbing, D., Kühnert, C. & West, G. B. Growth, innovation, scaling, and the pace of life in cities. *Proc. national academy sciences* **104**, 7301–7306 (2007).

70. Bonaventura, M., Aiello, L. M., Quercia, D. & Latora, V. Predicting urban innovation from the US Workforce Mobility Network. *Nature Humanities and Social Sciences Communications* **8** (2021).

71. Richterich, A. 'karma, precious karma!'karmawhoring on reddit and the front page's econometrisation. *J. Peer Prod.* **4**, 1–12 (2014).

72. Liben-Nowell, D., Novak, J., Kumar, R., Raghavan, P. & Tomkins, A. Geographic routing in social networks. *Proc. Natl. Acad. Sci.* **102**, 11623–11628 (2005).

73. Kuchler, T., Russel, D. & Stroebel, J. Jue insight: The geographic spread of covid-19 correlates with the structure of social networks as measured by facebook. *J. Urban Econ.* 103314 (2021).

74. Joglekar, S., Velupillai, S., Dutta, R. & Sastry, N. Analysing meso and macro conversation structures in an online suicide support forum. *arXiv preprint arXiv:2007.10159* (2020).

75. Calvillo, D. P., Ross, B. J., Garcia, R. J., Smelter, T. J. & Rutchick, A. M. Political ideology predicts perceptions of the threat of covid-19 (and susceptibility to fake news about it). *Soc. Psychol. Pers. Sci.* **11**, 1119–1128 (2020).

76. Venables, W. N. & Ripley, B. D. Random and mixed effects. In *Modern applied statistics with S*, 271–300 (Springer, 2002).

77. VIF: Variance Inflation Factor. https://www.rdocumentation.org/packages/regclass/versions/1.6/topics/VIF. Accessed: 2023-03-15.

78. Fatehkia, M., O'Brien, D. & Weber, I. Correlated impulses: Using facebook interests to improve predictions of crime rates in urban areas. *PloS one* **14**, e0211350 (2019).

79. Chetty, R. *et al.* Social capital i: measurement and associations with economic mobility. *Nature* **608**, 108–121 (2022).

80. Li, H., Hecht, B. & Chancellor, S. Measuring the monetary value of online volunteer work. In *Proceedings of the International AAAI Conference on Web and Social Media*, vol. 16, 596–606 (2022).

81. Newman, M. E., Barabási, A.-L. E. & Watts, D. J. *The structure and dynamics of networks.* (Princeton university press, 2006).

82. Leskovec, J. & Horvitz, E. Planetary-scale views on a large instant-messaging network. In *Proceedings of the 17th international conference on World Wide Web*, 915–924 (2008).

83. Fuchs, C. *Social media: A critical introduction* (Sage, 2021).

84. An, J., Quercia, D., Cha, M., Gummadi, K. & Crowcroft, J. Sharing political news: the balancing act of intimacy and socialization in selective exposure. *EPJ Data Sci.* **3**, 1–21 (2014).

85. Bobkowski, P. S., Jiang, L., Peterlin, L. J. & Rodriguez, N. J. Who gets vocal about hyperlocal: Neighborhood involvement and socioeconomics in the sharing of hyperlocal news. *Journalism Pract.* **13**, 159–177 (2019).

86. McCrae, R. R. & Costa Jr, P. T. The stability of personality: Observations and evaluations. *Curr. directions psychological science* **3**, 173–175 (1994).

87. Schurer, S., de New, S. & Leung, F. Do universities shape their students' personality? Tech. Rep., Institute of Labor Economics (IZA) (2015).

88. Bozarth, L. & Budak, C. Market forces: Quantifying the role of top credible ad servers in the fake news ecosystem. In *Proceedings of the International AAAI Conference on Web and Social Media*, vol. 15, 83–94 (2021).

89. Feingold, R. Fake news & misinformation policy practicum (2017).

90. Šćepanović, S., Aiello, L. M., Zhou, K., Joglekar, S. & Quercia, D. The healthy states of america: creating a health taxonomy with social media. In *Proceedings of the International AAAI Conference on Web and Social Media*, vol. 15, 621–632 (2021).

91. Hindman, D. B. The rural-urban digital divide. *Journalism & Mass Commun. Q.* **77**, 549–560 (2000).

92. Xu, R. *Corporate Fake News on Social Media*. Ph.D. thesis, University of Miami (2021).
